# Roles of the Cannabinoid System in the Basal Ganglia in Parkinson’s Disease

**DOI:** 10.3389/fncel.2022.832854

**Published:** 2022-02-21

**Authors:** Mengya Wang, Huayuan Liu, Zegang Ma

**Affiliations:** ^1^Department of Physiology, School of Basic Medicine, Institute of Brain Science and Disorders, Qingdao University, Qingdao, China; ^2^Department of Hepatobiliary Surgery, The Affiliated Qingdao Municipal Hospital of Qingdao University, Qingdao, China

**Keywords:** basal ganglia, Parkinson’s disease, CB1 receptors, CB2 receptors, cannabinoids

## Abstract

Parkinson’s disease (PD) is a neurodegenerative disease usually caused by neuroinflammation, oxidative stress and other etiologies. Recent studies have found that the cannabinoid system present in the basal ganglia has a strong influence on the progression of PD. Altering the cannabinoid receptor activation status by modulating endogenous cannabinoid (eCB) levels can exert an anti-movement disorder effect. Therefore, the development of drugs that modulate the endocannabinoid system may be a novel strategy for the treatment of PD. However, eCB regulation is complex, with diverse cannabinoid receptor functions and the presence of dopaminergic, glutamatergic, and γ-aminobutyric signals interacting with cannabinoid signaling in the basal ganglia region. Therefore, the study of eCB is challenging. Here, we have described the function of the cannabinoid system in the basal ganglia and its association with PD in three parts (eCBs, cannabinoid receptors, and factors regulating the cannabinoid metabolism) and summarized the mechanisms of action related to the cannabinoid analogs currently aimed at treating PD. The shortcomings identified from previous studies and the directions that should be explored in the future will provide insights into new approaches and ideas for the future development of cannabinoid-based drugs and the treatment of PD.

## Introduction

Parkinson’s disease (PD) is a common neurodegenerative disease that affects approximately 1% of the elderly population (Connolly and Lang, 2014). The main pathological feature of PD is the progressive loss and degeneration of the dopaminergic neurons projecting from the substantia nigra pars compacta (SNpc) to the striatum (Xu and Pu, 2016), with typical symptoms including deterioration of motor function, muscle rigidity, tremor, and postural instability (Dickson, 2018). According to current research, the pathogenesis of PD includes mitochondrial dysfunction, oxidative stress, abnormal Ca^2+^ regulation, neuroinflammation, and protein misfolding (More and Choi, 2015; Jiang and Dickson, 2018). Levodopa replacement therapy is the gold standard treatment for clinical PD. However, most patients experience serious side effects after long-term levodopa use, most notably levodopa-induced dyskinesia (LID) (Zhai et al., 2019). Current studies are dedicated to finding alternative drugs to levodopa that can reduce damage to nigrostriatal neurons without producing motor deficits (Utsumi et al., 2013). Recently, growing evidences showed that cannabinoids have neuroprotective and motor symptom-modulating properties, and clinical studies have observed a reduction in LID in patients with PD after smoking medical marijuana. Therefore, cannabinoids may be a promising candidate for the treatment of PD (Antonazzo et al., 2019; Cristino et al., 2020).

The endogenous cannabinoid system (ECS) is an important component of the basal ganglia neuromodulatory system, and its physiological functions include but are not limited to modulation of mood, cognition, motor, feeding, and pain (Pacher and Kunos, 2013). The ECS is composed of endogenous cannabinoids (eCBs), cannabinoid receptors, and various modulators responsible for the production, transport, and hydrolysis of cannabinoids (Lu and Mackie, 2016). The most prominent eCBs found to date are anandamide (AEA) and 2-arachidonoylglycerol (2-AG) (Gulyas et al., 2004; Ivanov et al., 2015). AEA and 2-AG are synthesized in different ways. There are three main pathways that mediate AEA synthesis. First, N-acyl-phosphatidylethanolamine-phospholipase D can release AEA by hydrolysis of N-acylphosphatidylethanolamine (NAPE). Second, α- and β-hydrolase domain-containing 4 and glycerophosphodiesterase 1 act continuously on NAPE to ultimately produce AEA (Muccioli, 2010). Third, phospholipase Cβ (PLCβ) hydrolyze N-arachidonoyl-phosphatidyl-ethanolamine, and the hydrolysis product can be dephosphorylated to AEA by PTPN22 (Di Marzo, 2011). 2-AG is synthesized in sn-2-arachidonic acid (AA)-containing diacylglycerol (DAG) membrane phospholipids by sequential hydrolysis of phospholipase C and DAG lipase (DAGL) α or β (Muccioli, 2010). In addition, the signaling pathway associated with phosphatidylinositol-4,5-diphosphate is also a mode of 2-AG synthesis (Maccarrone, 2017).

Degradation of eCBs is very rapid (Di Marzo et al., 1994; Maccarrone et al., 1998; Castillo et al., 2012) and is generally divided into two pathways—hydrolysis and oxidation (Castillo et al., 2012). The main hydrolytic enzymes of the first pathway are fatty acid amide hydrolase (FAAH) and monoacylglycerol lipase (MAGL), which are responsible for hydrolyzing AEA and 2-AG, respectively, and produce hydrolysis products such as ethanolamine or glycerol (Silvestri and Di Marzo, 2013). The main degrading enzyme of 2-AG is MAGL, a presynaptic localization enzyme that hydrolyzes 2-AG to AA and glycerol (Dinh et al., 2002; Tanimura et al., 2012). The enzyme responsible for the degradation of AEA is FAAH, which is located in the postsynaptic neuron cells (Egertova et al., 1998; Gulyas et al., 2004). The oxidative pathway is mainly regulated by cyclooxygenase-2 (COX-2), through which AA of eCBs can be partially oxidized (Kano et al., 2009). Lipoxygenase, acylglycerol kinase, and serine hydrolase α/β-hydrolase domains (ABHDs) can also be involved in the metabolism of eCBs (Kozak and Marnett, 2002; Bektas et al., 2005).

ECBs are lipophilic molecules, they are freely transported across the plasma membrane, but depend on concentration gradients to cross the plasma membrane with relatively low efficiency. Thus, AEA membrane transporter (AMT) is the main pathway for the entry of cannabinoids from the extracellular compartment to the intracellular compartment. Although AMT has not been cloned, its activity has been demonstrated in several neurons and in peripheral cells, and it has been validated in isolated brain tissue and synaptosomes from humans, mouse, and rat (Hillard et al., 1997; Maneuf et al., 1997; Maccarrone et al., 1998, 1999, 2000; Ruiz-Llorente et al., 2004). In addition, most studies thus far have demonstrated that AEA transport requires assistance via AMT, but there evidence suggests that AMT can also transport 2-AG (Hillard et al., 1997; Maccarrone et al., 1998, 2000; Rakhshan et al., 2000). Therefore, AMT can be considered the main cannabinoid transport protein.

The physiological effects of eCBs are mainly mediated by cannabinoid receptor (CB) 1 and/or CB2, which are widely distributed in the central nervous system, mostly in the basal ganglia, cortex, cerebellum, and hippocampus (Glass et al., 1997; Castillo et al., 2012; Jordan and Xi, 2019). In the brain, 2-AG levels are 170-fold higher than AEA, and 2-AG is a full agonist that activates Cannabinoid Receptor 1 (CB1) and Cannabinoid Receptor 2 (CB2) receptors, reducing cognitive flexibility and inhibiting responsiveness (Fagundo et al., 2013). Whereas AEA is a partial agonist of cannabinoid receptors and prefers to activate CB1 receptors (Sugiura et al., 2000; Soni et al., 2017; Baggelaar et al., 2018), contributing to increased cognitive flexibility and decision-making ability (Fagundo et al., 2013). In addition to these two receptors, recent studies have identified several other cannabinoid receptors, such as transient receptor potential vanilloid-1 (TRPV-1), G protein receptor-55 (GPR55), and peroxisome proliferator-activated receptors (PPARs) (Pertwee et al., 2010). The distribution of these cannabinoid receptors, their neuromodulatory mechanisms, and the role they play in neurodegenerative diseases have been a major target of research on the cannabinoid system.

## Distribution of Cannabinoids Receptors in the Basal Ganglia

**FIGURE 1:**
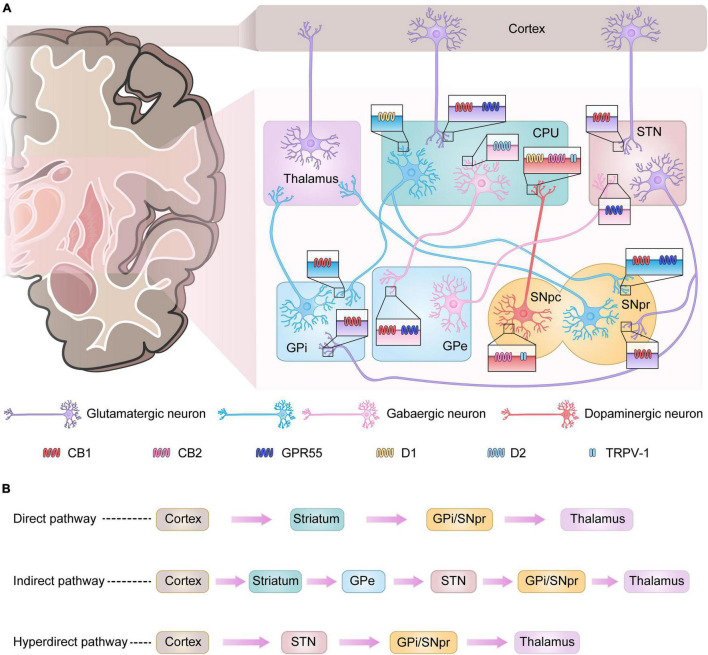
Basal ganglia signaling pathways and associated receptor localization. **(A)** Signals from the cerebral cortex affect the output nuclei (GPi and SNpr) via direct or indirect pathways via the basal ganglia input nuclei (CPU), which in turn regulate thalamic motor function. In Parkinson’s disease (PD), the loss of dopaminergic neurons leads to enhanced activity in the indirect pathway and diminished activity in the direct pathway, with more output of the inhibitory neurotransmitter gamma-aminobutyric acid (GABA) from the GPi and SNpr, which depresses the motor thalamus, leading to the development of PD. Some receptors present in the basal ganglia play an important role in the regulation of signaling. CB1 receptors are mainly found in GABAergic and glutamatergic neurons. GPR55 is present in the nuclei of GPe, CPU, STN, and SNpr. CB2 receptors and TRPV-1 are mainly present in dopaminergic neurons originating from the SNpc. **(B)** Three major pathways of basal ganglia signaling.

The basal ganglia are a group of gray matter nuclei in the subcortex of the brain, consisting of the striatum, globus pallidus, subthalamic nucleus (STN), and substantia nigra (Lanciego et al., 2012). The basal ganglia are involved in the regulation of motor function primarily through the cortico–basal ganglia–thalamo–cortical loop connection (Haber and Calzavara, 2009; Figure 1A). In this loop, the striatum is the afferent region of the basal ganglia, receiving fibers from cortical glutamatergic projections and then transmitting cortical motor signals to the efferent region of the basal ganglia—globus pallidus internus (GPi)/substantia nigra pars reticulata (SNpr)—via both direct and indirect pathways. GPi/SNpr is connected to the thalamus, through which the signal is finally returned to the cortex to induce the corresponding motor activity (DeLong and Wichmann, 2007). The only projection neurons of the striatum are medium-sized spiny neurons (MSNs) that express types 1 and 2 dopamine receptors (D1 and D2, respectively). Dopaminergic neurons originating from the SNpc project to the striatum, releasing dopamine that binds to D1 and D2 on MSNs for the direct and indirect pathways, respectively (DeLong and Wichmann, 2007). Dopamine acting on these two pathways inhibits GPi/SNpr and, thus, excites the thalamus, causing motor effects (Fernandez-Ruiz et al., 2010b). In PD, dopaminergic neurons become deficient and dopamine levels decrease, leading to an imbalance in the direct and indirect pathways of the basal ganglia, which then over-activate the GPi/SNpr and inhibit the thalamus, resulting in reduced motor cortical activity (Bezard et al., 2001). In addition, in recent years it has been proposed that a hyperdirect pathway projecting directly from the cerebral cortex to the STN is a relevant pathway for the basal ganglia to exert motor regulation and may have an influence on the development of PD (Nambu et al., 2002; Diesburg and Wessel, 2021; Figure 1B).

Numerous studies have shown that the ECS is involved in neural signaling in the basal ganglia. Radiographic autoradiography, immunohistochemistry, and immunoblotting have demonstrated a large number of CB1 and CB2 receptor-binding sites in the basal ganglia circuits, such as the striatum, substantia nigra, and globus pallidus (Munoz-Arenas et al., 2015). CB1 receptors are expressed in glutamatergic neurons in the corticostriatal and axon terminals of gamma-aminobutyric acid (GABA)-ergic neurons projecting to GPi/SNpr in the striatum (Kofalvi et al., 2005; Calabresi et al., 2014). Some studies have confirmed the localization of CB1 receptors in the glutamatergic nerve terminals innervating the STN and GPi/SNpr, while CB1 receptors are also expressed in the terminals of the glutamatergic corticostriatal neurons (Benarroch, 2007). However, some studies have shown that CB1 receptors are expressed not only in axon terminals but also in the cytosol (Fitzgerald et al., 2012). Recent studies have shown that CB2 receptors are also expressed in the central nervous system. Experimental studies in animals have demonstrated the expression of CB2 receptors in the striatum, globus pallidus, dopaminergic neurons in the ventral tegmental area (VTA), substantia nigra, and basal thalamus (Sierra et al., 2015; Zhang H. Y. et al., 2015; Gomez-Galvez et al., 2016; Sanchez-Zavaleta et al., 2018; Canseco-Alba et al., 2019; Jordan and Xi, 2019). The latest studies suggested that CB2 receptors are expressed in the cytosol and axon terminals of nigrostriatal dopaminergic neurons and that CB2 receptor expression is found in the SNpr of neonatal rats (Suarez et al., 2009; Lopez-Ramirez et al., 2020). However, CB2 receptors are believed to be mainly distributed in glial cells for neuroinflammation-related regulation (Amenta et al., 2012). In addition to the classical cannabinoid receptors, TRPV-1 is expressed in both cytosolic and axonal terminals of nigrostriatal dopaminergic neurons in the basal ganglia and in tyrosine hydroxylase-positive neurons in the dense part of the substantia nigra (Mezey et al., 2000; Cristino et al., 2006; Marinelli et al., 2007).

## Role of Cannabinoid Receptor 1 Receptors in Parkinson’s Disease

The main cannabinoid receptors are CB1 and CB2 receptors, which are a class of G protein-coupled receptor (GPCR) superfamily, coupled to inhibitory G proteins. The CB1 receptor was successfully cloned from a rat cerebral cortex cDNA library in 1990 using an oligonucleotide probe for members of the GPCR. Since the AC/cyclic adenosine monophosphate (cAMP) cascade signaling pathway inhibited by the CB1 receptor is able to control the activity of multiple cellular functions (Iannotti et al., 2016), and can regulate the electrical activity of neurons (Elphick and Egertova, 2001; Wilson and Nicoll, 2001), the CB1 receptor has been associated with a variety of neurological diseases and their development. Recent studies have found that CB1 receptors can regulate the transmission and metabolism of various neurotransmitters in PD lesions, thus regulating abnormal signaling in PD, In addition, CB1 receptors have modulatory effects on neuroinflammation, oxidative stress, excitotoxicity, neuroregeneration, and changes in cortical striatum plasticity in PD.

### Cannabinoid Receptor 1 Receptors Influence Parkinson’s Disease Progression by Regulating Neurotransmission

The main effects of CB1 receptors on PD has long been inconclusive due to the complex distribution of CB1 receptors in the basal ganglia and their modulation by multiple factors. There are four prevailing views.

#### Regulatory Role of Cannabinoid Receptor 1 Receptors in the Course of Parkinson’s Disease

**FIGURE 2:**
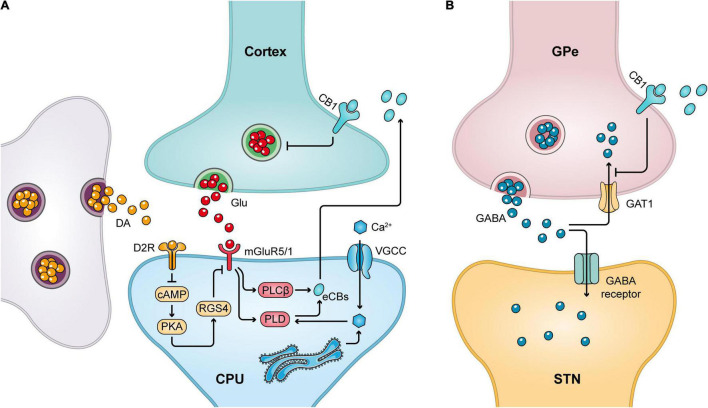
CB1 receptor-mediated retrograde signaling mechanism. **(A)** Release of Glu from the cortical striatal glutamatergic neurons can stimulate the release of eCBs from postsynaptic neurons, which in turn acts on CB1 receptors in the presynaptic membrane to inhibit presynaptic Glu release. Meanwhile, dopaminergic neurons can promote cannabinoid production by decreasing postsynaptic RGS4 phosphorylation levels. **(B)** Stimulation of the presynaptic CB1 receptor in GPe inhibits the reuptake of GABA by GABA transporter protein 1 (GAT1), thereby increasing local GABA levels.

As mentioned earlier, CB1 receptors are present in MSNs innervating GPi/e and SNpr. The effects of these CB1 receptors on nerve conduction are mediated by the retrograde synaptic messenger function of eCBs and can be explained by the release of postsynaptic neuronal eCBs that activate CB1 receptors on presynaptic axons, thereby reducing the release of the associated neurotransmitter (Wilson and Nicoll, 2002; Martin et al., 2008). For example, by stimulating presynaptic CB1 receptors in the terminals of corticostriatal glutamatergic neurons, Glu release can be reduced (Brotchie, 2003; van der Stelt and Di Marzo, 2003; Figure 2A). Similarly, activation of CB1 receptors present in the basal ganglia GPi/SNpr reduces Glu release from STN afferent neurons and GABA release from striatal afferent neurons. Conversely, stimulation of presynaptic CB1 receptors in the GPe reduces the reuptake of GABA from striatal afferent nuclei to increase local GABA levels (Brotchie, 2003; van der Stelt and Di Marzo, 2003; Benarroch, 2007; Figure 2B). Thus, CB1 receptors play an important role in regulating locomotion *in vivo*.

In PD, CB1 receptors tend to be overexpressed and activated in the basal ganglia. For example, in the brains of velvet monkeys treated with 1-methyl-4-phenyl-1, 2, 3, 6-tetrahydropyridine (MPTP), the number of striatal CB1 receptors is increased (Lastres-Becker et al., 2001), and the level of mRNA encoding CB1 receptors is increased in the striatum of rats treated with reserpine (Silverdale et al., 2001). In the indirect pathway, activation of CB1 receptors reduces the reuptake of GABA in striatal efferents to GPe, leading to accumulation of GABA in the synaptic gap, resulting in more inhibition of GPe by striatal GABAergic neurons and weakening GPe activity. This causes an increased release of Glu transmitters from the STN and inducing GPi/SNpr nuclei to release more GABA transmitters, thus further inhibiting the motor thalamus. Besides, in the direct pathway, GABA release by the D1 signaling pathway is reduced due to CB1 receptor effects. Both these effects promote the development of PD (Sieradzan et al., 2001). Therefore, cannabinoid antagonists may be able to slow the progression of Parkinson’s disease, and this conjecture is consistent with the results of several pharmacological experiments—the cannabinoid receptor antagonist rimonabant enhanced the locomotor activity of the dopamine receptor agonist quinpirole in normal rats and reserpinized rats and increased the locomotor activity in mice pre-administered with Δ9- tetrahydrocannabinol (Δ9-THC) (Giuffrida et al., 1999; Di Marzo et al., 2000; Huang et al., 2009).

However, in a clinical trial in which rimonabant was used in combination with levodopa, no anti-parkinsonian effect was found (Mesnage et al., 2004). This result may be due to the effect of levodopa masking the effect of rimonabant, or the dosage of rimonabant could be too low (Mesnage et al., 2004). However, based on the results of other studies, we propose a hypothesis that although in GPi/SNpr, overactivation of CB1 receptors produces two effects, one reducing the release of input terminal transmitters from striatal GABAergic neurons and another increasing the release of input terminal transmitters from STN glutamatergic neurons (Sanudo-Pena et al., 1999), the cannabinoid system is more effective in reducing glutamate release than in reducing GABA release (Sanudo-Pena et al., 1998). Therefore, in this region, activation of CB1 receptors could instead enhance motor thalamic activity and antagonizing CB1 receptors no longer has an antiparkinsonian effect.

In summary, CB1 receptors have different effects in different regions of the basal ganglia. In the striatum and in the output ganglia of GPi/SNpr, enhanced CB1 receptor transmission may alleviate PD, whereas if CB1 is activated in GPe, it exacerbates PD. Whether activation of CB1 receptors is beneficial in alleviating Parkinson’s disease requires a more comprehensive consideration and stronger clinical trial evidence.

#### Interaction Between Cannabinoid Receptor 1 Receptors and Dopamine Receptors

The regulation of CB1 receptors also interacts with the complex regulatory mechanisms of dopamine receptors, making it a complex physiological activity. Thus, many experiments exploring the role of CB1 agonists and antagonists and their application in PD have yielded inconsistent data (Papa, 2008). For example, by examining the effects of the cannabinoid agonists CP 55,940 and Win-55,212-2 on the rotational behavior induced by SKF 38393 (D1 dopamine receptor agonist), it was found that both cannabinoid agonists attenuated the behavior induced by SKF 38393, but the same response was not observed when the D2 agonist quinpirole was used. However, in the reserpine-treated rat model, Win-55,212-2 inhibited the effect of D2 agonists, but did not decrease the effect of D1 dopamine receptor agonists (Maneuf et al., 1997). In these experiments, although the effects of cannabinoid agonists on different dopamine agonists differed, it can be suggested that cannabinoid agonists antagonize the effects of dopamine drugs.

Further studies have revealed that dopaminergic signaling and eCBs signaling are bidirectional connections. Indeed, D1 and D2 are co-localized with CB1 receptors in the striatum in the striatonigral and globus pallidus GABAergic neural pathway (Brotchie, 2003; van der Stelt and Di Marzo, 2003; Benarroch, 2007; Martin et al., 2008; Mathur and Lovinger, 2012). The interaction of D1/D2 with CB1 receptors at the level of the G protein/adenylate cyclase signaling mechanism has been reported (Giuffrida et al., 1999; Meschler and Howlett, 2001; Munoz-Arenas et al., 2015). D2 and CB1 receptors may share a pool of G proteins, and D1 dopamine receptor-mediated activation of adenylate cyclase can be completely blocked by CB1 stimulation. Co-activation of D1 and CB1 receptors led to a decrease in adenylate cyclase and GABA transmitters in striatal direct projection neurons, which further led to an increase in SNpr neuronal activity and inhibition of locomotion. Conversely, co-activation of D2 and CB1 receptors increases adenylate cyclase, which in turn enhances indirect pathway effects and activates STN glutamatergic neurons, leading to motor inhibition (Glass et al., 1997; Brotchie, 2003; van der Stelt and Di Marzo, 2003; Calabresi et al., 2014; Munoz-Arenas et al., 2015). This coexistence of macromolecular complexes composed of functional receptor units with different biochemical properties is known as receptor heteromers. The presence of CB1–D2 receptor heteromers was confirmed using a FRET study by Marcellino et al. (2008). These studies have suggested that the activation effect of CB1–D2 receptor heteromers is different from that of individual receptors (Marcellino et al., 2008). Meanwhile, in the ventral striatum, the overlapping subcellular distribution of CB1 and D2 receptor immunoreactivity was confirmed by double-labeled electron microscopic analysis, demonstrating the presence of CB1–D2 receptor heteromers in the striatum (Pickel et al., 2006). The emergence of these heterodimeric phenomena suggests that CB1 receptors may inhibit movement in a coactivating manner with dopamine receptors and influence Parkinson’s disease progression.

Activation of CB1 receptors is mutually regulated with dopamine receptor signaling and there is a reciprocal regulatory relationship between dopamine secretion and eCBs release. First, dopamine activation of D2-like receptors modulates NAPE-phospholipase D and FAAH activity, stimulating the synthesis of AEA in the striatum while inhibiting its degradation, ultimately leading to an increase in AEA levels (Giuffrida et al., 1999; Beltramo et al., 2000). Such regulatory activity increases the role of eCBs in striatal D2 receptors activation and is an inhibitory feedback mechanism that limits the effects of dopamine. However, some studies have shown that after stimulation of dopamine, the AEA produced can enhance the activation effect of D2 receptors, thus appearing as a synergistic effect of D2 and CB1 receptors (Alonso et al., 1999; Meschler et al., 2000; Nava et al., 2000). Subsequent studies have shown that the inhibitory effect of GABA delivery via the D2 receptor pathway can be blocked or partially prevented by cannabinoid receptor antagonists, suggesting that CB1 may also be a downstream effector of D2 (Centonze et al., 2004).

In conclusion, the interaction between the dopamine and endocannabinoid systems is complex, and the current findings suggest that CB1 receptor activation can antagonize the effects of dopamine receptor agonists on the one hand, and inhibit movement by co-activating with dopamine receptors on the other. These findings suggest that CB1 receptor activation may exacerbate Parkinson’s disease progression. However, CB1 receptors may also act as a downstream effector of D2 receptors, enhancing the effects of D2 receptor activation and attenuating Parkinson’s symptoms. Thus, CB1 receptors have effects on the progression of PD, but more experimental evidences are still needed for the specific effects and mechanisms of influence.

#### Effect of Cannabinoid Receptor 1 Receptors Stimulation on Levodopa-Induced Dyskinesia

Currently, the most effective method of relieving PD symptoms is DA replacement therapy; however, long-term treatment with levodopa usually leads to changes in motor response called dyskinesias or abnormal involuntary movements (AIMs) (Nutt, 2000; Obeso et al., 2000). The anti-parkinsonian effect via cannabinoid antagonists suggests that cannabinoid agonists antagonize the LID effects induced by dopaminergic drugs. The specific mechanism may be that stimulation of CB1 receptors decreases striatal glutamate release, inhibits the effects of D1 receptor stimulation, and increases GABA levels in the GPi to reduce LID (Brotchie, 2003). This hypothesis is supported by experimental evidence that the CB1 agonist Win-55,212-2 produces an anti-motor effect in neurotoxin 6-hydroxy dopamine (6-OHDA)-injured rats and that the CB1 antagonist rimonabant reverses this effect (Ferrer et al., 2003). Furthermore, in the MPTP-constructed PD model, the combination of the cannabinoid receptor agonist nabilone with levodopa resulted in a smaller chance of dyskinesia compared to levodopa alone (Fox et al., 2002). The first clinical, double-blind, placebo-controlled trial of cannabinoids showed that CB1 receptor agonism significantly reduced the occurrence of LID (Sieradzan et al., 2001).

Ahmed et al. found that D2 dopaminergic receptors activation may reduce glutamate release in the striatum of patients with PD and alleviate LID symptoms, and this effect was dependent on CB1 receptor activation (Ahmed et al., 2011). The mechanism is the activation of D2 dopaminergic receptors, which promotes eCBs production, which in turn activates striatal CB1 receptors (Beltramo et al., 2000).

Taking together, CB1 receptors interact with D1 and D2 to exert a modulating effect on LID, and it is tentatively determined that CB1 receptor agonists can alleviate LID symptoms. However, the cannabinoid antagonist rimonabant has also been found to reduce the occurrence of LID in MPTP-treated marmoset experiments (van der Stelt et al., 2005). It is possible that such results occur because CB1 receptors are not effectively blocked and that only a complete blockade of CB1 receptors in a given situation may determine the role of CB1 antagonists, for example, the use of CB1 receptor antagonists at low doses in moderate PD or when PD progresses to an advanced stage (Fernandez-Espejo et al., 2005; Gonzalez et al., 2006). Although these experimental results may require further confirmation of the final results due to different experimental conditions as well as animal selection, they indicate that the effectiveness of CB1 antagonists in alleviating the LID is unclear.

#### Cannabinoid Receptor 1 Receptors Modulate Cortico-Striatal Plasticity

Sustained stimulation of MSN synapses located in the striatum induces long-term depression (LTD) and long-term potentiation of synaptic transmission efficacy. This synaptic plasticity plays an important role in the formation of neuronal circuits that maintain normal movement and learning. eCBs are involved in the formation of LTD synapses that connect cortical neurons to the striatum and, therefore, play a crucial role in maintaining normal neural circuits at striatal synapses. Recent studies have suggested that corticostriatal glutamatergic synaptic plasticity remodeling is a key factor in improving behavioral function in PD (Petzinger et al., 2013). Reduced glutamatergic regulation in patients with PD leads to over-activation of the corticostriatal glutamatergic pathway, resulting in dyskinesia (More and Choi, 2015). While activation of CB1 receptors inhibits corticostriatal glutamatergic synaptic transmission (Gerdeman and Lovinger, 2001), and reshapes synaptic structural plasticity (Gerdeman et al., 2002).

Retrograde messenger function mediated by eCBs is essential for the production of corticostriatal LTD, also known as eCB–LTD (Mathur and Lovinger, 2012). eCB–LTD occurs specifically in MSNs in the striatal indirect pathway and is regulated by D2 (Gerdeman et al., 2002). The mechanism is that activation of the D2 dopamine receptors inhibits cAMP/protein kinase A (PKA) activity and reduces the phosphorylation of its downstream protein, RGS4 (Cerovic et al., 2013), which in turn increases the production of eCBs (Lerner and Kreitzer, 2012). Cannabinoids are transported to the synaptic gap and bind to CB1 receptors on the presynaptic membrane, leading to depolarization-induced de-excitation, which then induces eCB-LTD (Cerovic et al., 2013; Figure 2A). In support of the above, the LTD between MSN synapses in the indirect pathway was abolished in the experimental model of PD (Di Filippo et al., 2008; Pisani et al., 2011). This deficit can be restored using D2 dopamine receptor agonists such as quinpirole or URB597, an inhibitor of FAAH (Kreitzer and Malenka, 2007). Furthermore, in another experimental model using PD, URB597, and quinpirole exhibited reduced convulsions and increased motility. Thus, restoration of cortical striatal synaptic eCB-mediated synaptic plasticity contributes to the improvement in motor symptoms in PD (Kreitzer and Malenka, 2007).

Taken together, activation of CB1 receptors helps to restore the formation of LTD synapses between cortical neurons and striatum, which in turn improves motor symptoms in PD.

### Cannabinoid Receptor 1 Receptors Activation Influence Parkinson’s Disease Progression

In addition to modulating PD through neurotransmission signaling, CB1 receptor activation can also modulate the neurological levels of cytokines to influence the progression of PD.

#### Modulation of Neuroinflammation by Cannabinoid Receptor 1 Receptors Stimulation

Neuroinflammation is a key pathological factor contributing to PD (Tiwari and Pal, 2017). Glial cells in the brain and various pro- or anti-inflammatory cytokines are the main factors that regulate neuroinflammation.

CB1 receptors play an important immunomodulatory role in the nervous system of animal models (Maresz et al., 2007). In a study exposing CB1 receptor-knockout and wild-type mice both injected with CB1 receptor agonists to stress, wild-type mice were found to reduce secretion of pro-inflammatory molecules (Zoppi et al., 2011). Further, dopaminergic neuronal damage induced by the neurotoxin MPTP can be alleviated by CB1 agonists by reducing microglial activation and decreasing the levels of pro-inflammatory factors such as interleukin (IL)-1β, tumor necrosis factor-α (TNF-α), and inducible nitric oxide synthase (iNOS) (Chung et al., 2011). In addition, the CB1-selective agonist ACEA exerted a protective effect on neurons in a model of lipopolysaccharide-induced inflammation (Vrechi et al., 2018). In addition to these effects, activation of CB1 receptors can regulate the release of neurotrophic factors and anti-inflammatory mediators. In a previous study, the effect of cannabinoids on CB1 receptors reduced the levels of TNF-α and IL-12 and promoted the release of IL-10, which facilitated the repair and regeneration of damaged neurons (Smith et al., 2000). In astrocytes, there is a primary brain glutamate transporter (excitatory amino acid transporter-2). CB1 receptor activation regulates the anti-inflammatory pathway L-PGDS/15d-PGJ2/PPARγ to promote the activation of this transporter. This activation indirectly inhibits nuclear factor-κB (NF-κB) activity and reduces the expression of iNOS and COX-2, thereby alleviating neuroinflammation. In conclusion, CB1 receptor activation plays an important role in suppressing the production of inflammatory mediators and reducing neuroinflammatory responses.

#### Effect of Cannabinoid Receptor 1 Receptors Stimulation on Oxidative Stress in Parkinson’s Disease

Oxidative stress levels are maintained at higher levels in the SNpc of the brains of patients with PD (Jenner, 2003). Oxidative stress levels were later assessed by further testing oxidized proteins and lipids in the brain tissue of patients with PD, and it was found that oxidative stress levels may have an impact on the progression of PD (Puspita et al., 2017). The current findings suggest that oxidative stress can lead to degeneration of nigrostriatal dopamine neurons in the basal ganglia of patients with PD and that a number of factors that contribute to changes in oxidative stress levels are responsible for neuronal damage, such as mitochondrial dysfunction, nitric oxide (NO) toxicity, excitotoxicity, and inflammatory responses (Bolanos et al., 1997; Schapira and Gegg, 2011; Blesa et al., 2015).

Studies have shown that activation of CB1 receptors can regulate oxidative stress in PD. In a rotenone-induced PD model, oxidative stress levels were reduced after the addition of cannabinoid analogs (Abdel-Salam et al., 2015). The CB1 receptor agonist Win 55,212-2 has also been shown to reduce lipid peroxidation in a model of MPTP-induced PD (Rangel-Lopez et al., 2015; Escamilla-Ramirez et al., 2017). In another MPTP-induced PD animal model experiment, activation of CB1 receptors reduced the pro-oxidative effects produced by microglia (Chung et al., 2011).

In addition, dysregulated levels of oxidative stress caused by PD are also associated with mitochondrial dysfunction in neuronal cells. This dysfunction leads to the accumulation of reactive oxygen species in cells, further exposing neuronal cells to oxidative damage and causing apoptosis (Mattson et al., 2008; Schapira, 2008). Recent studies have suggested that CB1 receptors are present in the mitochondrial membrane (Hebert-Chatelain et al., 2014). Some cannabinoids may improve mitochondrial dysfunction by activating CB1 receptors. For example, after using exogenous cannabinoids such as Δ9-THC to act on neurons in the mouse brain, they can activate CB1 receptors on mitochondrial membranes and reduce cAMP and PKA concentrations, which is beneficial for improving neuronal mitochondrial dysfunction (Benard et al., 2012). Evidence also suggests that Δ9-THC can repair damaged mitochondria by restoring the levels of proteins that maintain normal mitochondrial function. One of the important ways in which it acts is the regulation of the PPARγ pathway (Carroll et al., 2012; Zeissler et al., 2016). In conclusion, activation of CB1 receptors can directly regulate the level of oxidative stress in PD and also resist oxidative damage by improving mitochondrial function.

#### Modulation of Excitotoxicity by Cannabinoid Receptor 1 Receptors Stimulation

Excitotoxicity is a process in which glutamate receptors are excessively or chronically activated, resulting in Ca^2+^ overload and neuronal necrosis or apoptosis. In PD, glutamate release is facilitated by neuroinflammatory effects. The aggregation of α-synuclein due to PD increases glutamate-induced synaptic currents, thereby exacerbating glutamate-related excitotoxicity. In addition, activation of glutamate receptors located on microglia leads to the release of additional pro-inflammatory factors, further exacerbating neuroinflammation and oxidative stress, creating a vicious cycle of glutamate over-release (Ambrosi et al., 2014). As mentioned above, eCBs and their receptors regulate glutamate release through a retrograde signaling system at the synapse; thus, the ECS plays an important role in the treatment of diseases characterized by abnormal glutamate homeostasis. Reduced levels of glutamate release through activation of CB1 receptors can inhibit excitotoxicity in PD (Fernandez-Ruiz et al., 2010a; Ohno-Shosaku and Kano, 2014).

In addition, N-methyl-D-aspartate (NMDA) receptors cause Ca^2+^ influx by mediating the synaptic transmission of glutamate, leading to damaged energy metabolism in neurons (Doble, 1999). It has been reported that activation of CB1 receptors can reduce NMDA-induced Ca^2+^ influx (Liu et al., 2009). The CB1 receptor agonists Win-55,212-2 and AEA were found to increase the survival of rat neuronal cells, which was attributed to the reduction of Ca^2+^ release caused by NMDA over-activation by CB1 receptor agonists (Fagan and Campbell, 2014). In conclusion, inhibition of excessive activation of Ca^2+^ channels by activating postsynaptic CB1 receptors in NMDA receptor-containing neurons is also a mechanism through which CB1 receptors exert neuroprotective effects.

In addition to the above mechanism of effect, CB1 receptors can also inhibit excitotoxicity by modulating PKA and interfering with AMPA-type glutamate receptor transport capacity (Zhuang et al., 2005; Fagan and Campbell, 2014). Moreover, treatment with Win-55,212-2 also inhibits TNF-α-induced excitotoxicity (Zhao et al., 2010). Other studies have suggested that brain-derived neurotrophic factor (BDNF) may be critical for CB1-mediated protection against excitotoxicity (Khaspekov et al., 2004; Blazquez et al., 2015). In conclusion, these findings suggest that activation of CB1 receptors can inhibit the excitotoxicity of PD by a variety of mechanisms, mainly including retrograde signaling as well as NMDA receptor antagonism. This suggests that CB1 receptors have great potential in improving Parkinson’s symptoms.

#### Cannabinoid Receptor 1 Receptor Activation Promotes Neurogenesis

Neurogenesis was discovered 50 years ago and is a physiological process that generates new neurons in the adult forebrain. When neurons are damaged in the brain, the level of this neurogenesis is regulated to produce new neurons and migrate to the site of injury (Goncalves et al., 2008). Neuronal damage is usually accompanied in many neurodegenerative diseases. Therefore, new neuronal generation and the establishment of new neural connections are necessary to maintain normal neuronal function and improve disease symptoms (More et al., 2012; Fagan and Campbell, 2014).

Recent studies have found that some non-motor symptoms due to PD, such as depression, may be due to the dysfunction of neurogenesis in the brain (Marxreiter et al., 2013; Gyorfi et al., 2017). Adult neurogenesis defects have been detected in the brains of patients with PD (Bruck et al., 2004; Winner and Winkler, 2015). The mechanism may be due to the abnormal aggregation of wild-type α-synuclein affecting the function of neuronal populations in the striatum (Harding et al., 2002; McKeith et al., 2004). In addition, α-synuclein activates microglia to produce neuroinflammation, which directly affects neural stem cells, thus reducing neuronal regeneration potential (Das et al., 2011; Russo et al., 2011; Worlitzer et al., 2012).

In recent years, as the ECS has been studied in depth, there is increasing evidence that cannabinoid signaling plays a key role in regulating neurogenesis. CB1 and CB2 receptors and DAGLα have also been identified in neural stem cells, further suggesting that the cannabinoid system is involved in the regulation of neurogenesis (Prenderville et al., 2015; Rodrigues et al., 2017). Activation of CB1 receptors generally promotes neurogenesis; for example, the CB1-specific agonist ACEA induces neural stem cell differentiation and maturation and promotes neural regeneration (Compagnucci et al., 2013). Genetic deletion of CB1 receptors results in neurogenesis defects (Jin et al., 2004). Dentate gyrus proliferating cells were reduced by 50% in the animal model of the CB1CB1-/- genotype in previous studies (Jin et al., 2004; Kim et al., 2006). Besides, CB1 receptors are also associated with neurogenesis in excitotoxicity models (Aguado et al., 2007).

The reason for this effect may be that stimulation of CB1 receptors increases levels of BDNF, a neurotrophic factor that promotes adult neurogenesis and is often reduced due to PD (Khaspekov et al., 2004; Scharfman et al., 2005; Zuccato and Cattaneo, 2007). Continued in-depth study has suggested that CB1 receptors regulate elevated BDNF gene expression levels through activation of the phosphatidylinositol 3-kinase (PI3K)/Akt/mammalian target of rapamycin complex 1 (mTORC1) pathway. BDNF can also promote neuronal sensitivity to the ECS (Maison et al., 2009). In addition, activation of CB1 receptors reduces Ca^2+^ inward flow from cerebellar granule cells through the voltage-activated Ca^2+^ channels, decreases the efficiency of NO synthesis by neuronal nitric oxide synthase, and antagonizes the neurotoxic effects of NO, thus promoting neurogenesis (Hillard et al., 1999; Kim et al., 2006; Marchalant et al., 2009). The neuroinflammatory response caused by PD also affects nerve regeneration, and activation of CB1 receptors can suppress the chronic inflammatory response and restore the level of nerve regeneration, besides, related experiments have also confirmed that CB1 receptors promote neural stem cell proliferation through the IL-1 signaling pathway (Garcia-Ovejero et al., 2013).

In summary, activation of CB1 receptors may promote nerve regeneration by increasing BNDF levels and decreasing inflammation levels, and contribute to the repair of damaged nerves in PD; however, there are some different experimental findings; for example, a continuous 3-week oral administration of incremental doses of Δ9-THC was found to have no effect on cell proliferation in the dentate gyrus of mice. Further, oral administration of a static dose of Δ9-THC for 6 weeks reduced cell proliferation, but had no effect on overall neurogenesis in mice (Wolf et al., 2010). Therefore, the relationship between CB1 receptors and nerve regeneration needs to be further explored.

In conclusion, the current studies suggest that activation of CB1 receptors inhibits neuroinflammation, alleviates oxidative stress, reduces excitotoxicity, and promotes neural regeneration, thus having a better effect on PD. Activation of CB1 receptors also alleviates LID and restores synaptic plasticity. However, in terms of activation of CB1 receptors on neurotransmission, activation of CB1 receptors in GPe has the potential to exacerbate PD and increase dyskinesia. Therefore, it is necessary to investigate the specific role of CB1 receptors in the basal ganglia on PD and their mechanisms in depth.

## Role of Cannabinoid Receptor 2 Receptors in Parkinson’s Disease

CB2 receptor is the second cannabinoid receptor that is discovered in humans (Munro et al., 1993). Studies have shown that CB2 receptors are distributed in neurons in the cortex, striatum, amygdala, hippocampus, and VTA (Gong et al., 2006; Onaivi et al., 2006; Zhang et al., 2014). Therefore, activation of CB2 receptors could influence the progression of PD by regulating neurotransmission and neuronal function. Furthermore, some findings have shown that in the brain, CB2 receptors are abundantly distributed in activated astrocytes and microglia (Stella, 2010). Thus, they have a crucial role in regulating neuroinflammation (Benito et al., 2008; Price et al., 2009).

### Role of Cannabinoid Receptor 2 Receptors in the Regulation of Dopaminergic Neuronal Activity

Recent evidence suggests that there is signaling modulation involving CB2 receptors in neuronal cells in the basal ganglia under brain injury conditions. In particular, CB2 receptors are more distributed in pale globus pallidus projection neurons (Fernandez-Ruiz et al., 2007; Lanciego et al., 2011). In addition, a higher number of CB2 receptors were also found in human tissues in dopaminergic neurons present in the nigrostriatal pathway, suggesting that CB2 receptors regulate dopaminergic neuronal activity by affecting interneuronal signaling (Garcia et al., 2015). Although the function of CB2 receptors in nigrostriatal neurons has not been demonstrated, CB2 receptors on dopaminergic neurons located in the VTA have been shown to modulate the excitability of dopaminergic neurons in mouse models; therefore, CB2 receptors in nigrostriatal neurons may also have this function (Zhang et al., 2014). The presence of reduced CB2 receptors in nigrostriatal dopaminergic neurons in PD also suggests that nigrostriatal degeneration may be associated with changes in CB2 receptor function (Garcia et al., 2015).

However, in general, the distribution of CB2 receptors in nerve cells is limited, and their role is relatively limited. Studies related to the regulation of nerve conduction in which CB2 receptors are involved have not revealed the mechanism of action of CB2 receptors. Therefore, further studies on the effects of CB2 receptors on nerve conduction in the basal ganglia are needed.

### Effect of Cannabinoid Receptor 2 Receptors Stimulation on Neuronal Function

CB2 receptor activation also reduces the number of myeloperoxidase-producing astrocytes and increases antioxidant enzyme activity, thereby reducing the level of excessive oxidation in animal models of PD (Chung et al., 2016; Javed et al., 2016). And some cannabinoids have intrinsic structural antioxidant properties that contribute to their effectiveness as antioxidants (Javed et al., 2016). In addition, the CB2 receptor agonist Hu-308 has been found to attenuate the excitotoxicity induced by quinolinic acid injections (Palazuelos et al., 2009).

CB2 receptors are also thought to affect the proliferation of neural progenitor cells (Palazuelos et al., 2006). The use of the CB2 receptor agonist Hu-308 activates the PI3K/Akt/mTORC1 pathway, exerting a pro-proliferative effect on neuronal cells (Palazuelos et al., 2012). In addition, CB2 receptors have been found in neural stem cells and can regulate the proliferation of neural precursor cells (Molina-Holgado et al., 2007; Garcia-Ovejero et al., 2013). It has also been found that activation of these CB2 receptors is also beneficial in alleviating the decrease in neurogenesis induced by age, as neuronal cell proliferation and partial restoration of neurogenesis levels occurred in the brains of aged animals after treatment with the CB2 receptor agonist Win-55,212-2 (Goncalves et al., 2008). Furthermore, CB2 receptors facilitate the repair of brain damage by promoting neuroblast proliferation, formation of new neurons, and repair of damaged areas (Bravo-Ferrer et al., 2017).

In conclusion, CB2 receptors can alleviate oxidative damage and excitotoxicity in PD, promote neural regeneration, and thus slow down the progression of Parkinson’s disease. However, the regulatory mechanisms of CB2 receptors on various cytokines secreted by the nervous system are still in the exploratory stage, and more experiments are needed to investigate their effects on the function of the basal ganglia nervous system.

### Effects of Cannabinoid Receptor 2 Receptors Stimulation on Neuroinflammation

Neuroinflammation is an important factor in the development and progression of disease in patients with PD. An over-activated inflammatory response leads to the death of dopaminergic neurons, and glial cells play an important role in the regulation of neuroinflammation (Tiwari and Pal, 2017). Microglia in the substantia nigra of the brain are abnormally activated in patients with PD (More et al., 2013).

Recent studies have shown that cannabinoids can exert anti-inflammatory effects through CB2 receptors (Klegeris et al., 2003; Esposito et al., 2007; Garcia-Arencibia et al., 2007; Stella, 2010). Many studies on drugs that can activate the action of CB2 have demonstrated the ameliorative effect of CB2 receptor activation on neuroinflammation. For example, β-caryophyllene (BCP) inhibited the activation of p38 mitogen-activated protein kinase (MAPK) and NF-κB pathways through the activation of CB2R, reduced microglial activation, and decreased the release of inflammation-associated cytokines (Segat et al., 2017). A CB2 receptor agonist, JWH-133, has been shown to exert neuroprotective effects by increasing glutamate uptake and decreasing pro-inflammatory cytokine levels. Mice overexpressing CB2 receptors exhibited similar anti-inflammatory effects, whereas CB2-/- mice were susceptible to the induction of neuroinflammatory responses (Zoppi et al., 2014). In addition, JWH-133 also counteracts MPTP-induced neurodegeneration and inhibits microglial activation (Chung et al., 2016). Another CB2 agonist, HU-308, has also been shown to reduce neuroinflammation in a study by Gomez-Galvez et al. (2016) who showed that HU-308 reduced LPS-induced inflammatory effects by activating CB2 receptors. In a mouse model of traumatic brain injury, the CB2 receptor agonist GP-1a was reported to promote M2 macrophage polarization while reducing M1 macrophages, and this effect was reversed by the CB2 receptor antagonist AM630 (Braun et al., 2018).

Another group also found that treatment of nigrostriatal tyrosine hydroxylase-positive neurons with the CB2 receptor agonist JWH-015 attenuated MPTP-induced pro-inflammatory effects and reduced microglial activation in a PD model (Price et al., 2009). Later studies have further revealed that JWH-015 causes a decrease in NF-κB activity in LPS-activated BV-2 cells, thus exerting a neuroprotective effect (Ribeiro et al., 2013). However, this inhibition could not be reversed by CB2 or CB1 antagonists, suggesting that the specific role of CB2 receptors requires further investigation.

Targeting CB2 receptors induces neuroprotective effects because CB2 receptors are mainly distributed in brain glial cells and neural precursor cells, their expression is rapidly enhanced after activation, and they have a wide range of effects, directly regulating inflammation-related cells (Amenta et al., 2012; Chung et al., 2016). Moreover, because of the less distribution of CB2 receptors in nerve cells, they do not produce central side effects such as abnormal nerve conduction similar to CB1 receptor activation. Therefore, modulating glial cell activation through CB2 receptors to further inhibit abnormally active neuroinflammation in the basal ganglia would be an effective approach for treating PD.

## Effects of Other Cannabinoid Receptors on Parkinson’s Disease

Although CB1 and CB2 receptors mediate the main processes by which cannabinoids act, the role of other cannabinoid receptors in the basal ganglia is gradually being revealed. Among them, TRPV-1 has been more intensively studied; it is present in sensory neurons and dopaminergic neurons of the nigrostriatal pathway in the basal ganglia circuit. It constitutes a regulatory mechanism that interacts with CB1 receptors in the regulation of dopaminergic neuronal activity (Mezey et al., 2000; Micale et al., 2009). AEA not only activates the CB1 receptor but also acts as a partial agonist of the TRPV-1 receptor (Sawzdargo et al., 1999; Zygmunt et al., 1999; Di Marzo et al., 2001). The way AEA acts is typical of the interaction between TRPV-1 and CB1 receptor activation. In particular, TRPV-1 and CB1 receptors may play opposite roles in LID, and after increasing levels of AEA with URB597, co-administration of the TRPV-1 antagonist capsazepine was more effective in alleviating levodopa-induced AIMs. Similarly, the CB1 agonist Win-55,212-2, which inhibits TRPV-1 receptors, has the same effect on AIMs as the combination of CB1 agonist and TRPV-1 antagonist. TRPV-1 receptor antagonists in combination with FAAH inhibitors can exert better anti-motility disorder effects (Morgese et al., 2007). These experiments revealed that eCB activation of the TRPV-1 receptors may antagonize the anti-motor impairment effects of the CB1 receptors it activates (Caterina et al., 1997; Morgese et al., 2007). However, in other studies, TRPV-1 agonists were found to not only inhibit the anti-movement disorder effect of OEA, but also improve movement disorder by decreasing the expression of movement disorder-related molecules (Gonzalez-Aparicio and Moratalla, 2014). Therefore, further studies are needed to prove the role of TRPV-1 in levodopa-induced AIMs.

TRPV-1 receptors play different roles in different neurons. TRPV-1 receptors, present in striatal dopaminergic neurons in the substantia nigra, are activated to reduce the synthesis and secretion of dopamine in striatal dopaminergic neuron terminals (Mezey et al., 2000; de Lago et al., 2004). In contrast, TRPV-1 receptors present in glutamatergic neuronal cells in the substantia nigra, when activated, can instead stimulate dopamine release (Marinelli et al., 2003, 2007). CB1 receptor antagonists may also elevate dopamine levels by blocking TRPV-1 receptors on dopaminergic neurons in the nigrostriatal, producing an effect against bradykinesia in PD. Thus, blocking TRPV-1 receptors at the terminals of dopaminergic neurons may have a therapeutic effect in the treatment of PD, but this effect decreases gradually with neuronal death and loss of TRPV-1 receptors due to disease progression (Lastres-Becker et al., 2005).

TRPV-1 receptors can also regulate neuronal function (van der Stelt et al., 2001; Veldhuis et al., 2003). It was found that capsaicin-activated TRPV-1 inhibits oxidative stress and exerts neuroprotective effects in a 6-OHDA-induced animal model of PD (Martinez-Pinilla et al., 2019). However, excessive activation of TRPV-1 leads to elevated intracellular Ca^2+^ levels and disrupts mitochondrial function, which in turn promotes neurotoxicity (Kim et al., 2005). Moreover, the TRPV-1 antagonist rimonabant has been shown to promote neuronal cell proliferation in the brain of mice. However, this was not observed in TRPV-1-knockout mice (Jin et al., 2004). Therefore, further investigation is needed to clarify whether the effect of TRPV-1 on neuronal cell function is two sided.

Taken together, antagonizing TRPV-1 receptors may alleviate LID, whereas activating TRPV-1 receptors may attenuate oxidative damage in PD, and TRPV-1 receptors can affect dopamine secretion. However, these findings are controversial. More experiments are still needed to explore the effect of TRPV-1 receptors on PD progression.

Another possible cannabinoid receptor is GPR55, which is highly expressed in the striatum and has been found to exert a neuroprotective effect in a rat model of excitotoxic injury (Sawzdargo et al., 1999; Ryberg et al., 2007; Celorrio et al., 2017). In addition, GPR55 may also interact with cannabinoids and may be regulated by CB1 receptors (Ulrich et al., 2014; Zheng et al., 2017). Recent studies have shown that cannabidiol (CBD) can exert neuromodulatory effects through the regulation of GPR55 (Morano et al., 2020). However, the mechanism by which GPR55 exerts its anti-inflammatory effects is unclear (Kallendrusch et al., 2013).

In conclusion, in addition to the above mentioned cannabinoid receptors, other cannabinoid receptors exist in the basal ganglia, such as GPR18, GPR119, and PPARs (Ferrisi et al., 2021), which together constitute the cannabinoid system in the basal ganglia, but the roles of these receptors have not been clarified. Therefore, it is necessary to explore the functions of these cannabinoid receptors in order to refine the mechanism of action of the cannabinoid system.

## Factors Regulating Endogenous Cannabinoids Metabolism in the Basal Ganglia in Parkinson’s Disease

The concentration levels of eCBs in the basal ganglia also directly influence cannabinoid-related effects; thus, the various factors that regulate cannabinoid metabolism are also important influences on the function of cannabinoids in the basal ganglia. Overall, cannabinoid metabolism has been found to include the synthesis, transport, and catabolism of cannabinoids. The main studies have focused on two cannabinoid hydrolases, FAAH and MAGL, which have been shown to have therapeutic effects in PD through their inhibition, thereby increasing eCBs levels in the basal ganglia region.

### Fighting Depression and Anxiety Symptoms Caused by Parkinson’s Disease

In addition to the typical symptoms of movement disorders, patients with PD often experience psychiatric symptoms such as depression and anxiety. FAAH-related inhibitors can improve depressive symptoms by modulating monoaminergic signaling and improving the function of the hypothalamic–pituitary–adrenal axis (Ogawa and Kunugi, 2015). For example, some FAAH inhibitors, such as URB597, URB694, and ST4070, can improve blood corticosterone levels under stressful conditions, thus alleviating depression or anxiety (Hill et al., 2009; Carnevali et al., 2015; Marco et al., 2015; Ogawa and Kunugi, 2015; Danandeh et al., 2018). However, the MAGL inhibitor JZL184 increased corticosterone levels, although the MAGL inhibitor had a significant antidepressant effect. The mechanism could be attributed to the removal of the inhibition of synaptic function by astrocytes and restoration of the glutamatergic or GABAergic synapses to normal activity, while MAGL inhibitors also activate mTOR signaling and can promote neurogenesis in brain as well as maintenance of intersynaptic LTD (Aliczki et al., 2013; Zhong et al., 2014; Zhang Z. et al., 2015; Wang et al., 2017). These mechanisms can alleviate psychiatric symptoms. In addition, since the main effect of MAGL inhibitors is to increase the levels of 2-AG, which mainly activates CB2 receptors and regulates neuroinflammation, they seem to contribute to the improvement of depressive symptoms associated with neuroinflammation. JZL184 has been shown to decrease the levels of inflammatory factors such as IL-6 and TNF-α in a lipopolysaccharide-induced neuroinflammation model (Kerr et al., 2013).

### Cannabinoid Hydrolase Inhibitors in Parkinson’s Disease

In PD models, URB597 ameliorates MPTP-induced neuroinflammation, decrease microglia inflammatory response, reduce dopaminergic neuron death, and alleviate PD symptoms. Johnston et al. (2011) further explored the efficacy of combining L-DOPA and URB597 and found that URB597 reduced L-DOPA-induced hyperactivity symptoms by 52%. This result suggests that the combination of URB597 and L-DOPA can eliminate the side effects of L-DOPA and improve the efficacy of L-DOPA in the treatment of PD (Johnston et al., 2011). In addition, other studies have performed a comparative analysis of the role of FAAH and MAGL inhibitors in PD. By selecting KML29 (inhibitor of MAGL) and PF-3845 (inhibitor of FAAH) for separate experiments in disease models, it was found that KML29 treatment attenuated striatal dopaminergic neuronal damage in MPTP/probenecid mice and increased glial derived neurotrophic factor expression levels, but it did not affect the increased CB2 receptor expression induced by MPTP use. However, PF-3845 showed neither a definitive protective effect nor enhanced the expression of CB2 receptors. Therefore, MAGL inhibitors are more effective for the treatment of PD (Pasquarelli et al., 2017).

However, FAAH inhibitors are uniquely positioned to counteract excitotoxicity by activating the neuroprotective effects of CB1 receptors without producing side effects similar to those of other CB1 agonists (Janero et al., 2009). AM5206, a reversible inhibitor of FAAH, has shown neuroprotective effects in both *in vivo* and *ex vivo* experiments. AM5206 exhibited a protective effect on synaptic proteins in an animal model of excitotoxic injury (Naidoo et al., 2011). In addition, several recent studies further support the idea that FAAH inhibitors play a role in preventing excitotoxicity in various excitotoxicity models (Naidoo et al., 2011; Aguilera-Portillo et al., 2019).

Furthermore, knockdown of FAAH promotes the proliferation of neuronal cells, whereas inhibition of 2-AG production causes complete cessation of neuronal cell proliferation in the subventricular zone (SVZ) of mouse (Aguado et al., 2005; Goncalves et al., 2008). Further studies revealed that in MPTP- and 6-OHDA-induced disease models, URB597 exerted neuroprotective effects through mechanisms such as reducing lipid peroxidation levels and decreasing protein carbonyls and reactive oxygen species production (Pelicao et al., 2016; Escamilla-Ramirez et al., 2017).

In conclusion, the reason why cannabinoid hydrolase inhibitors possess significant neuroprotective effects is mainly dependent on the fact that the eCBs have neurogenic and neuroprotective functions. Correspondingly, reducing the expression of eCBs synthase inhibits neuroprotective effects. For example, knockdown of DAGLα enzyme resulted in a substantial decrease in 2-AG and AEA levels in the brain and reduced the rate of neuronal cell proliferation in the brain in a previous study (Gao et al., 2010). Therefore, controlling cannabinoid levels by modulating cannabinoid-related regulatory enzymes and, thus, treatment of the disease, is a novel therapeutic strategy. This approach produces fewer side effects and has more applications than the modulatory effects of cannabinoid receptor agonists.

## Effects of Cannabinoid Drugs on Parkinson’s Disease

The cannabinoid system in the basal ganglia can be regulated not only by the corresponding receptors and enzymes. Some phytocannabinoids can also act on the cannabinoid receptors in the basal ganglia, thus affecting the cannabinoid system. The representative phytocannabinoids are Δ9-THC and CBD, of which CBD is the most promising drug for PD due to its low side effects and it does not produce anxiety and other psychiatric symptoms (Dalton et al., 1976; Zuardi et al., 2006; Ibeas Bih et al., 2015). The current study found that Δ9-THC and CBD can exert neuroprotective effects by affecting inflammation and acting as antioxidants (Pertwee, 2005).

Δ9-THC restored levels of mitochondrial function-related proteins by activating the PPARγ pathway, and resisted MPTP-induced cytotoxicity in *in vitro* experiments, reduced reactive oxygen species production in the PD (Carroll et al., 2012; Zeissler et al., 2016). Δ9-THC also prevents dopaminergic neuronal damage in a model of 6-OHDA-induced neuroinflammation (Lastres-Becker et al., 2005). In an *in vitro* experimental model of PD, neuroprotective effects were observed after Δ9-THC treatment in SH-SY5Y cells (Carroll et al., 2012). Furthermore, Δ9-THC reduces excitotoxicity induced by ouabain by activating CB1 receptors (Veldhuis et al., 2003), and Δ9-THC activates CB1 receptors on neuronal mitochondrial membranes, which in turn regulates cAMP concentration and the enzymatic activity of PKA and complex I, contributing to the repair of neuronal mitochondrial function (Benard et al., 2012).

Similarly, CBD counteracts dopaminergic neuronal damage in an animal model of 6-OHDA-induced neuroinflammation (Lastres-Becker et al., 2005). The roles played by CBD in PD generally include neuroprotective effects, such as antioxidant, anti-inflammatory, and neurogenesis-promoting effects, and modulation of LID. On the one hand, CBD can inhibit microglia activation and pro-inflammatory molecule release through PPARγ receptor-mediated anti-inflammatory effects. The specific mechanism of this effect is related to the inhibition of p38 MAP kinase and NF-κB (Esposito et al., 2007; Scuderi et al., 2014). In addition, CBD can affect the migration and recruitment of microglia and slow the propagation of inflammation (Walter et al., 2003). On the other hand, CBD can increase the neuroprotective effects of cannabinoids by inhibiting FAAH or reducing the transport rate of eCBs to increase AEA levels in the brain (Bisogno et al., 2001; Leweke et al., 2012). Some studies have also confirmed that in the MPP^+^-induced PD model, CBD could induce cell differentiation while inhibiting caspase-3 activation and increasing nerve growth factor levels, promoting neurogenesis (Santos et al., 2015).

The antioxidant effect of CBD works through a mechanism that is not dependent on CB1 or CB2 receptors, which allows to avoid the increased dyskinesia associated with the activation of CB1 receptors (Fernandez-Ruiz, 2009; Fernandez-Ruiz et al., 2013). Several studies have confirmed this idea; for example, CBD has an antioxidant activity in *in vitro* cellular assays, but has no affinity for CB1 or CB2 receptors (Campillo and Paez, 2009). Subsequent studies have demonstrated that the antioxidant mechanism of CBD may involve the regulation of certain intracellular antioxidant factors. In a 6-OHDA-induced nigrostriatal DA depletion model, CBD was found to increase the transcript levels of the antioxidant enzyme Cu, Zn-superoxide dismutase, which is a key enzyme for defense against oxidative stress (Garcia-Arencibia et al., 2007). Related studies have revealed that CBD upregulates Nrf2 transcription in BV-2 microglia and increases downstream antioxidant-related enzymes, including heme oxygenase-1 and glutathione transferase. The increase in these enzymes exerts significant antioxidant and counteracting cytotoxic effects (Juknat et al., 2012, 2013).

CBD can also be used to improve LID produced in PD treatment, in one study, CBD was found to improve LID in five patients with PD compared to standard drug therapy and correlated with the dose of CBD taken (Consroe et al., 1986). And the relevant mechanism, as shown in previous studies, revealed that levodopa metabolism is dependent on 5-hydroxytryptamine (5-HT) neurons and that selective agonists of 5-HT1 receptors have been found to reduce LID in animal models of PD. Moreover, previous studies have shown that CBD can modulate 5-hydroxytryptaminergic neurotransmission (Magen et al., 2010; Zanelati et al., 2010; Espejo-Porras et al., 2013). Related *in vitro* experiments have shown that CBD activated 5-HT1A (Rock et al., 2012). In addition, the exercise-improving effects of CBD are not affected by CB1 antagonists, but they are antagonized by selective 5-HT1A receptor antagonists (Espejo-Porras et al., 2013). These results suggest that CBD can affect LID by modulating 5-HT1A receptors. In addition, in experiments exploring whether the application of CBD can alleviate levodopa-induced AIMs, CBD in combination with TRPV-1 antagonists was found to better treat LID (Dos-Santos-Pereira et al., 2016). In recent years, as the mechanism of action of CBD has been studied in depth, new targets of CBD action have been identified, such as the GPR6 orphan receptors expressed mainly in striatal and globus pallidus neurons (Lobo et al., 2007). GPR6 receptor knockdown causes a decrease in AIMs and an increase in dopamine levels. This suggests that inhibition of GPR6 receptor expression can exert anti-motor impairment effect (Oeckl et al., 2014). Some studies suggest that CBD may work as an inverse agonist of the GPR6 receptor by binding to GPR6 through its pentyl side chain and free hydroxyl group, which in turn reduces GPR6-mediated recruitment of β-arrestin2. Therefore, GPR6 is considered as a new target for CBD (Laun and Song, 2017; Laun et al., 2019). Taken together, CBD may also alleviate LID by inhibiting the GPR6 receptor (Patricio et al., 2020).

However, there are still many questions about the specific effects of phytocannabinoids and their mechanisms of action, which need to be addressed. For example, in a randomized controlled trial, a mixture of oral Δ9-THC and CBD showed no improvement in LID (Carroll et al., 2004). And Wolf et al. (2010) found that oral administration of certain doses of Δ9-THC can reduce neuronal cell proliferation, besides, this study have shown that long-term CBD administration also reduced neuronal cell proliferation, which seems to contradict our current mainstream understanding. A clinical trial also found that patients with PD receiving CBD did not show better outcomes (Chagas et al., 2014). The reason for this result may be due to the small sample size and the short duration of treatment in this trial, but these unexpected results l suggest that there are still many questions to be answered about whether phytocannabinoids can be used in the treatment of PD. Further research is needed to explore the role and specific mechanisms by which phytocannabinoids play in the basal ganglia and PD. Overall, phytocannabinoids are an emerging and promising drug to be used as an adjunct to levodopa in the treatment of PD.

## Conclusion and Outlook

In recent years, the exploration of the role and mechanisms of cannabinoids in PD has gradually increased, and new targets and functions of cannabinoids in the nervous system have been gradually revealed. In the progression of PD, the function of cannabinoids has gradually become an important influence that cannot be ignored, especially the ECS in the basal ganglia, which plays an important role in the compensatory regulation of PD, and the response to cannabinoid analogs and complex functional regulation. In this paper, based on the composition of the cannabinoid system in the basal ganglia, we introduced the functions of endogenous cannabinoids, the distribution and mode of action of cannabinoid-related receptors and the effects of modulators targeting the cannabinoid system on Parkinson’s disease, summarized the roles and mechanisms of the cannabinoid system in antioxidation, inhibition of inflammation and promotion of neuroprotection. Cannabinoids were found to have a wide range of potential applications in PD treatment, not only to combat the symptoms of dyskinesia in PD but also to improve some PD-related psychiatric symptoms or side effects of drugs such as LID.

However, the exploration of the specific mechanisms of action and the targets of action of cannabinoids is still not comprehensive enough, and some findings are still controversial, especially the exact effects of CB1 receptor activation on PD and the mechanism of action of many cannabinoid-related drugs, which require further experimental evidence. Current research methods on these aspects also have some shortcomings, such as the differences between artificially induced PD and naturally occurring PD in mice and rats, which do not fully reflect the role of the cannabinoid system in the pathogenesis of PD. In addition, there are mechanisms that have not been studied in depth enough to fundamentally reveal the mechanism of cannabinoid action. For example, for some new cannabinoid receptors, relevant experimental studies are still lacking. For the CB2 receptor, its specific distribution in the basal ganglia and the role it plays are still unclear. And the cannabinoids themselves lack a preparation process, and the use of concentrations and purity is not uniform, among other shortcomings, which also affects the credibility of the experimental research and cause an overdose or underdose, resulting in experimental failure.

Therefore, further studies on the cannabinoid system in the basal ganglia are needed to adjust experimental methods, establish more reliable animal models, and improve the preparation process of the cannabinoid drugs used for experiments to ensure reliable experimental results. Concurrently, more comprehensive study designs need to be conducted to integrate the effects of cannabinoids in all aspects, explore the main pathways in which the cannabinoid system acts and other secondary pathways, and fully consider the combined effects of individual receptor activation before the mechanisms of the effects of the cannabinoid system in the basal ganglia on PD may finally be revealed.

## Author Contributions

MW: writing–original draft, writing review and editing. HL: writing–review and editing. ZM: writing–review and editing, project administration, funding acquisition, and Supervision. All authors contributed to the article and approved the submitted version.

## Conflict of Interest

The authors declare that the research was conducted in the absence of any commercial or financial relationships that could be construed as a potential conflict of interest.

## Publisher’s Note

All claims expressed in this article are solely those of the authors and do not necessarily represent those of their affiliated organizations, or those of the publisher, the editors and the reviewers. Any product that may be evaluated in this article, or claim that may be made by its manufacturer, is not guaranteed or endorsed by the publisher.

## Abbreviations

PD: Parkinson’s disease
eCB: endogenous cannabinoid
COX-2: cyclooxygenase-2
ECS: endogenous cannabinoid system
AEA: anandamide
2-AG: 2-arachidonoylglycerol
NAPE: acylphosphatidylethanolamine
PLCβ: phospholipase Cβ
DAG: diacylglycerol
SNpc: substantia nigra pars compacta
LID: levodopa-induced dyskinesia
AA: arachidonic acid
DAGLα: diacylglycerol lipase alpha
FAAH: fatty acid amide hydrolase
MAGL: monoacylglycerol lipase
ABHD: α/β -hydrolase domain
AMT: anandamide membrane transporter
CB: cannabinoid receptor
TRPV-1: transient receptor potential vanilloid-1
GPR55: G protein-coupled receptor 55
PPAR: peroxisome proliferator-activated receptor
STN: subthalamic nucleus
GPi: globus pallidus internus
GPe: globus pallidus externa
SNpr: substantia nigra pars reticulata
MSN: medium spiny neuron
D1: type 1 dopamine receptor
D2: type 2 dopamine receptor
GABA: gamma-aminobutyric acid
VTA: ventral tegmental area
GPCR: G protein-coupled receptor
MAPK: mitogen-activated protein kinase
MPTP: 1-methyl-4-phenyl-1,2,3,6-tetrahydropyridine
6-OHDA: 6-hydroxy dopamine
AIM: abnormal involuntary movement
CBD: cannabidiol
LTD: long-term depression
cAMP: cyclic adenosine monophosphate
PKA: protein kinase A
TNF- α: tumor necrosis factor- α
iNOS: inducible nitric oxide synthase
IL: interleukin
NF- κ B: nuclear factor- κ B
NO: nitric oxide
NMDA: N-methyl -D-aspartate
SVZ: subventricular zone
BDNF: brain-derived neurotrophic factor
PI3K: phosphatidylinositol 3-kinase
mTORC1: mammalian target of rapamycin complex 1
BCP: β-caryophyllene
Δ 9-THC: Δ 9- tetrahydrocannabinol
CBD: cannabidiol
5-HT: 5-hydroxytryptamine.
